# Design, Physicochemical Characterization, and In Vitro Permeation of Innovative Resatorvid Topical Formulations for Targeted Skin Drug Delivery

**DOI:** 10.3390/pharmaceutics14040700

**Published:** 2022-03-24

**Authors:** Victor H. Ruiz, David Encinas-Basurto, Bo Sun, Basanth Babu Eedara, Sally E. Dickinson, Georg T. Wondrak, H. -H. Sherry Chow, Clara Curiel-Lewandrowski, Heidi M. Mansour

**Affiliations:** 1 Department of Pharmacology and Toxicology, The University of Arizona College of Pharmacy, Tucson, AZ 85721, USA; vruiz@pharmacy.arizona.edu (V.H.R.); dencinas@pharmacy.arizona.edu (D.E.-B.); bosun@pharmacy.arizona.edu (B.S.); bbeedara@pharmacy.arizona.edu (B.B.E.); wondrak@pharmacy.arizona.edu (G.T.W.); 2 Center for Translational Science, Florida Interational University, Port St. Lucie, FL 34987, USA; 3 University of Arizona Cancer Center, University of Arizona, Tucson, AZ 85724, USA; sdickinson@uacc.arizona.edu (S.E.D.); schow@arizona.edu (H.-H.S.C.); ccuriel@arizona.edu (C.C.-L.); 4 Department of Pharmacology, The University of Arizona College of Medicine, Tucson, AZ 85724, USA; 5 Department of Medicine, Division of Hematology and Oncology, The University of Arizona College of Medicine, Tucson, AZ 85724, USA; 6 Department of Medicine, Division of Dermatology, The University of Arizona College of Medicine, Tucson, AZ 85724, USA; 7 BIO5 Institute, University of Arizona, Tucson, AZ 85724, USA; 8 Department of Medicine, Division of Translational & Regenerative Medicine, The University of Arizona College of Medicine, Tucson, AZ 85724, USA

**Keywords:** resatorvid (TAK-242), nonmelanoma skin cancers, topical drug delivery, HaCaT human skin cell line, NHEK normal primary human skin cells, Strat-M synthetic membrane, Epiderm^TM^ 3D human skin, in vitro cell viability, retention, flux, diffusion, Franz cell, hydrogel, polyethylene glycol (PEG), Pluronic^®^ poloxamer triblock copolymer, carbomer gel, propylene glycol (PG), hyaluronic acid (HA) gel, emulsion, cream

## Abstract

Nonmelanoma skin cancers (NMSCs) are the most common malignancies worldwide and affect more than 5 million people in the United States every year. NMSC is directly linked to the excessive exposure of the skin to solar ultraviolet (UV) rays. The toll-like receptor 4 (TLR4) antagonist, resatorvid (TAK-242), is a novel prototype chemo preventive agent that suppresses the production of inflammation mediators induced by UV exposure. This study aimed to design and develop TAK-242 into topical formulations using FDA-approved excipients, including DermaBase^TM^, PENcream^TM^, polyethylene glycol (PEG)-400, propylene glycol (PG), carbomer gel, hyaluronic acid (HA) gel, and Pluronic^®^ F-127 poloxamer triblock copolymer gel for the prevention of skin cancer. The physicochemical properties of raw TAK-242, which influence the compatibility and solubility in the selected base materials, were confirmed using X-ray powder diffraction (XRPD), differential scanning calorimetry (DSC), hot-stage microscopy (HSM), Raman spectroscopy, and attenuated total reflectance Fourier-transform infrared (ATR-FTIR) spectroscopic analysis. The permeation behavior of TAK-242 from the prepared formulations was determined using Strat-M^®^ transdermal diffusion membranes, and 3D cultured primary human-derived epidermal keratinocytes (EpiDerm^TM^). Despite TAK-242′s high molecular weight and hydrophobicity, it can permeate through reconstructed human epidermis from all formulations. The findings, reported for the first time in this study, emphasize the capabilities of the topical application of TAK-242 via these multiple innovative topical drug delivery formulation platforms.

## 1. Introduction

Skin cancers, including melanoma and nonmelanoma, are the most common malignancies worldwide [1]. Nonmelanoma skin cancers (NMSCs) represent a group of skin cancers that include basal cell carcinoma (BCC) and squamous cell carcinoma (SCC). NMSCs affect more than 5 million people in the United States every year [2]. Exposure to environmental ultraviolet (UV) radiation is the major risk factor for the development of NMSCs [3]. Acute or chronic exposure of the skin to UV radiation induces the development of inflammation, oxidative stress, DNA damage, epigenetic changes in skin cells, and suppression of immune sensitivity, which together trigger the development of cutaneous malignancies [4]. Due to the high burden associated with SCC, the need to identify effective therapeutic prevention strategies is of primary importance. 

The toll-like receptor 4 (TLR4), an innate immune response receptor, has been proven as a major driver of cutaneous inflammation in response to UV exposure [5,6]. Several studies have reported the overexpression of TLR4 in SCC [7,8]. The activation of TLR4 by ligand binding of pathogenic or endogenous stimuli stimulates the production of inflammatory transcription factors, such as nuclear factor-κB (NF-κB), activator protein-1 (AP-1), and interferon regulatory transcription factor-3 (IRF3), which regulate inflammatory signaling, as well as apoptosis, survival, and differentiation [9]. Thus, pharmacological inhibition of TLR4 is a promising strategy for the prevention of UV-induced NMSC. 

**Figure 1:**
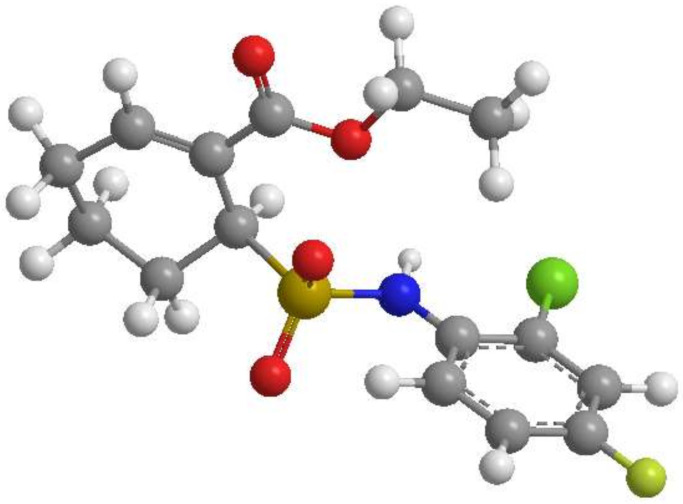
Resatorvid 3D molecular structures: (ChemDraw^TM^ Ver. 16.0; Cambridge Soft, Cambridge, MA, USA).

Resatorvid (TAK-242; Figure 1) is a selective TLR4-inhibitor that suppresses the production of lipopolysaccharide (LPS)-induced inflammation by irreversibly binding to a specific cysteine residue (Cys747) in the intercellular domain of TLR4 [7,10,11,12,13]. In preclinical studies, TAK-242 showed potent suppressive effects on the production of tumor necrosis factor-α (TNF- α), interleukin-1 (IL-1) and interleukin-6 (IL-6) in both murine [12] and porcine [14] models of sepsis. However, it failed to suppress the cytokine levels in patients with sepsis and shock or respiratory failure in phase III clinical studies (ClinicalTrials.gov: NCT00633477; NCT00143611) [15]. Other studies investigated the effect of TAK-242 as a neuroprotective agent in treating traumatic brain injury [16] and in suppressing UV-induced inflammatory signaling in the skin [7]. Janda, Burkett [7] observed that the topical application of TAK-242 on mouse skin potentiates UV-induced epidermal apoptosis. 

Additionally, a previous study by our group demonstrated that applying an acetone solution of TAK-242 (13 mM) successfully modulated UV-induced biomarkers in SKH-1 mice, and by using a standardized Franz cell system, we revealed that TAK-242 efficiently penetrates the stratum corneum and remains in the skin for at least 8 hr. While a significant amount of agent remained in the stratum corneum (tape strips 1–3), the amount of resatorvid in the lower epidermis (tape strips 4–12) remained consistent throughout the time course at an average of 5 g/cm^2^ [17]; we provided a first-of-its-kind study demonstrating that topical resatorvid increases skin residence time, reduces UV-induced AP-1 activation, and inhibits photocarcinogenesis in mice. The results of these studies indicate that TAK-242 is an emerging class of chemo preventive agent, and the topical application of TAK-242 is useful for both topical inhibition of UV-induced skin inflammation and, potentially, tumorigenesis.

This systematic and comprehensive study reports for the first time on multiple innovative topical drug delivery platforms for the incorporation of TAK-242 using various FDA-approved excipients (i.e., DermaBase^TM^, PENcream^TM^, polyethylene glycol-400 (PEG-400), propylene glycol (PG), carbomer gel, hyaluronic acid (HA) gel, and Pluronic^®^ F-127 poloxamer triblock copolymer gel) and using a non-sterile compounding technique for topical application to prevent skin cancer. Prior to the formulation, the solid-state nature of raw TAK-242, which influences the compatibility and solubility in the selected base materials, was determined using various analytical techniques. The in vitro permeation behavior of TAK-242 from the prepared formulations was determined using the synthetic Strat-M^®^ transdermal diffusion membrane and 3D cultured primary human epidermal keratinocytes (EpiDerm^TM^). In vitro cell viability as a function of drug dose for biocompatibility was performed on HaCaT skin cell line and on NHEK (normal human epidermal keratinocyte) primary cells. To the Authors’ knowledge, this study is the first to report these findings.

## 2. Materials and Methods

### 2.1. Materials

Resatorvid (TAK-242, molecular weight = 361.82 g/mol, C_15_H_17_ClFNO_4_S, >98% purity), shown in Figure 1, was purchased from APAC Pharmaceutical LLC (Columbia, MD, USA). Propylene glycol (PG, USP/FCC certified) was obtained from Fisher Scientific (Fair Lawn, NJ, USA). Pluronic^®^ F-127 (poloxamer triblock copolymer) powder was purchased from Sigma-Aldrich (St. Louis, MO, USA). Sodium hyaluronate (95%) powder was purchased from Acros Organics (Geel, Belgium). Carbomer 934P (Carbopol^®^ 934P) resin NF, polyethylene glycol 400 (PEG-400, PEG-8 NF, USP certified), and polyethylene glycol 600 (PEG-600, PEG-12 NF, USP certified) were purchased from Spectrum Chemical MFG Corp. (Gardena, CA, USA). PENcream^TM^ (versatile topical cream (oil-in-water type) for Rx compounding) was purchased from Humco (Texarkana, TX, USA). DermaBase^TM^ cream (oil-in-water emulsion base) was purchased from Perrigo (Minneapolis, MN, USA). Hydranal^®^-Coulomat AD and resazurin sodium salt were purchased from Sigma-Aldrich (St. Louis, MO, USA). 

Human keratinocytes cells (HaCaT, AddexBio^®^ T0020001) were purchased from AddexBio (San Diego, CA, USA). The Dulbecco’s modified Eagle’s medium (DMEM), Optimized 1X was obtained from AddexBio (San Diego, CA, USA). Fetal Bovine Serum (FBS), Pen-Strep, and Fungizone were obtained from Gibco^®^ by Life Technologies (Thermo Fisher Inc, Waltham, MA, USA). Normal human epidermal keratinocytes (NHEKs^®^), which are unique primary adult cell lines, and their growth medium (NHEK-GM^®^) were both purchased from MatTek Life Sciences (Ashland, MA, USA). EpiDerm^TM^, a 3D tissue model consisting of normal human-derived epidermal keratinocytes and its EpiDerm^TM^ special growth medium were both purchased from MatTek Life Sciences (Ashland, MA, USA). The Strat-M^®^ synthetic membrane (Transdermal Diffusion Test Model, 47 mm) was purchased from Millipore Sigma (Danvers, MA, USA).

### 2.2. Physicochemical Characterization of Raw Resatorvid

#### 2.2.1. Scanning Electron Microscopy (SEM) and Energy-Dispersive X-ray (EDX) Spectroscopy

The particle size and surface morphology of raw resatorvid were obtained using scanning electron microscopy (SEM) (FEI, Brno, Czech Republic). The samples were sprinkled onto the double-sided adhesive carbon tabs (Ted Patella, Inc. Redding, CA, USA), which were adhered to aluminum stubs and coated with a 7 nm thin film of gold palladium alloy, using an Anatech Hummer 6.2 (Union City, CA, USA) sputtering system at 20 µA for 90 s under an argon plasma. The electron beam with an accelerating voltage of 30 kV was used at a working distance of approximately 9–12 mm. The SEM images were collected at various magnifications.

EDX was performed using ThermoNoran System Six (Thermo Scientific, Waltham, MA, USA) at an accumulation voltage of 30 keV; the spot size was increased until a dead time of 20–30 s was obtained. 

#### 2.2.2. Particle Sizing and Size Distribution by SEM Image Analysis

Using SEM micrographs, the mean size, standard deviation, and size range of the particles were determined digitally using SigmaScan^TM^ Pro 5.0.0 (Systat, Inc., San Jose, CA, USA). Representative micrographs of raw resatorvid at 400–5000× magnification were analyzed by measuring the diameter of at least 60 particles per sampling.

#### 2.2.3. X-ray Powder Diffraction (XRPD) 

The powder crystallinity of raw resatorvid was determined by X-ray powder diffraction (XRPD). XRPD patterns of raw resatorvid were collected at room temperature with a PANalytical X’pert diffractometer (PANalytical Inc., Westborough, MA, USA) equipped with a programmable incident beam slit and an X’celerator detector. The X-ray radiation used was Ni-filtered Cu Kα (45 kV, 40 Ma, and λ = 1.5444 Å). Measurements were taken between 5° and 89.9° (2θ) with a scan rate of 2°/min. The powder samples were loaded on a zero-background silicon sample holder. 

#### 2.2.4. Differential Scanning Calorimetry (DSC) 

The thermal analysis and phase transition behavior of raw resatorvid was determined using a TA Q1000 differential scanning calorimeter (DSC) (TA Instruments, New Castle, DE, USA) equipped with T-Zero^®^ technology, RSC90 automated cooling, and an auto sampler. The instrument was previously calibrated with indium (99.9% purity). Approximately 1–3 mg of raw resatorvid was weighed into anodized hermetic aluminum DSC pans and hermetically sealed with aluminum lids using a T-Zero^®^ hermetic press (TA Instruments, New Castle, DE, USA). An empty, hermetically sealed aluminum pan was used as a reference. UHP nitrogen gas was used as the purging gas at a flow rate of 40 mL/min. The samples were heated from 0 °C to 250 °C at a scanning rate of 5 °C/min. All measurements were carried out in triplicate. 

#### 2.2.5. Hot-Stage Microscopy (HSM)

Thermo-microscopic changes of the raw resatorvid were observed and recorded using a Leica DMLP cross-polarized microscope (Wetzlar, Germany) equipped with a Mettler FP 80 central processor heating unit and Mettler FP82 hot stage (Columbus, OH, USA). Raw resatorvid was sprinkled onto a glass slide and heated from 25 °C to 250 °C at a heating rate of 5 °C/min. The images were digitally captured using a Nikon Coolpix 8800 digital camera (Nikon, Tokyo, Japan) under 10× optical objective and 10× digital zoom.

#### 2.2.6. Karl Fisher Titration (KFT)

The residual water content of raw resatorvid powder was quantified by coulometric KFT using a TitroLine 750 trace titrator (SI Analytics, Weilheim, Germany). In brief, 1 mL of 0.1% (*w*/*v*) of resatorvid in anhydrous methanol was added to the titration cell containing Hydranal^®^ Coulomat AD reagent. 

#### 2.2.7. Raman Spectroscopy 

The chemical homogeneity of raw TAK-242 was analyzed via Raman spectroscopy at 785 nm laser excitation using a Renishaw inVia Reflex (Gloucestershire, UK), using a 20× magnification objective on a Leica DM2700 optical microscope (Wetzlar, Germany). This system had a 2400 grooves/mm grating, with a slit width of 65 µm and a thermoelectrically cooled Master Renishaw CCD detector. The laser power was adjusted to achieve 5000 counts per second for the 520 cm^−1^ line of the internal Si reference. Raman spectra were acquired with 1% of laser power and 10 s of exposure for raw TAK-242 powder. Spectra were subjected to baseline correction before further analysis using Renishaw WiRE 3.4 software.

#### 2.2.8. Attenuated Total Reflectance Fourier-Transform Infrared (ATR-FTIR) Spectroscopy

The molecular fingerprint and presence of functional groups of raw resatorvid were analyzed via ATR-FTIR spectroscopy using a Nicolet Avatar 360 FTIR spectrometer (Varian, Inc., Palo Alto, CA, USA) equipped with a DTGS detector and a Harrick MNP-Pro (Pleasantville, NY, USA) attenuated reflectance (ATR) accessory. Each spectrum was collected at 32 scans at a spectral resolution of 2 cm^−1^ over the wavenumber range of 4000–400 cm^−1^. A background spectrum was carried out under the same experimental conditions. Spectral data were acquired with EZ-OMNIC software.

### 2.3. Preformulation of Resatorvid: Solubility and Compatibility

Seven platforms were analyzed for compatibility with resatorvid. The platforms were DermaBase^TM^ cream (cream and lotion), PENcream^TM^ (cream), PEG-400 solution, PG solution, 1% (*w*/*v*) carbomer 934P resin gel at a pH range of 6–7, 2% (*w*/*v*) sodium hyaluronate gel/serum, and 20% (*w*/*v*) Pluronic^®^ F127 gel. The maximum solubility of TAK-242 was determined at concentrations ranging from 0.1–5% *w*/*v* using phosphate-buffered saline (PBS, pH = 7.41), 0.9% normal saline (NS), ethanol, PEG-400, and PG as solvents. The purpose of dissolving TAK-242 at a series of concentrations was to determine the saturated solubility in different solvents by looking at any precipitates or turbidity within the solution. The following step was to test if TAK-242 could be directly incorporated into the platforms mentioned above via non-sterile compounding to determine if the drug would need to be pre-dissolved before incorporation. A determination of how much volume of PEG-400 or PG could be incorporated into the creams and gels without losing consistency while maintaining homogeneity was examined; 1 g of base (cream or gel) was weighed out and was compounded with PEG-400 or PG (500 µL–1000 µL). The drug was then dissolved in the appropriate determined volume of a compatible solvent and sonicated for 60 min; then, it was prepared via the non-sterile compounding technique, and the formulations were examined for physical appearance (consistency, homogeneity, visible precipitation, phase separation, and compatibility). The only formulation that was different was the Pluronic^®^ F127, as everything was mixed in as a liquid at 4 °C, and the solution would then solidify at room temperature (~37 °C) to create the gel, which was then mixed thoroughly until homogenous.

### 2.4. Preparation of Topical Formulations by Non-Sterile Compounding 

A total of eight formulations of resatorvid were prepared via the non-sterile compounding technique. The formulations were chosen based on drug compatibility and solubility in the base materials, PEG-400 and PG, after a series of test trials. TAK-242 was dissolved in 20 mL scintillation vials after water-bath sonication, and a glass slab (12×12, 1.5″ thick) was used for non-sterile compounding. 

The DermaBase^TM^ cream formulation was made at concentrations of 1.25% (*w*/*w*), 2.5% (*w*/*w*), and 5% (*w*/*w*). Resatorvid was dissolved in PEG-600 using a Branson 2800 sonicator (Ultrasonics LLC., Richmond, VA, USA). Once the drug was fully dissolved, the formulation was made via non-sterile compounding, which is a common technique used in pharmacies to create a medication in a clean environment but does not require the environment to be completely free of microorganisms. The PENcream^TM^ formulation and DermaBase^TM^ lotion were prepared with the same method. The solvent for resatorvid in the DermaBase^TM^ lotion was PG. 

The carbomer gel formulation was made at drug concentrations of 1.25% (*w*/*w*) and 2.5% (*w*/*w*). The gel base was made at a concentration of 1% (*w*/*v*) using carbomer 934P and milli-Q water. The pH was adjusted to 6–7 with triethanolamine. Resatorvid was dissolved in PEG-400 using a Branson 2800 sonicator. Once the drug was fully dissolved, the formulation was compounded via the non-sterile compounding technique. The hyaluronic acid gel/serum formulation was made at concentrations of 1.25% (*w*/*w*) and 2.5% (*w*/*v*). The gel/serum base was made at a concentration of 2% (*w*/*v*) using sodium hyaluronate (95%) and milli-Q water. Resatorvid was dissolved in PEG-400 using a Branson 2800 sonicator. Once the drug was dissolved, the formulation was compounded using a non-sterile technique. The Pluronic^®^ gel formulation was made at a concentration of 1.25% (*w*/*v*) and 2.5% (*w*/*v*). The gel base was made using Pluronic^®^ (F127) at a concentration of 20% (*w*/*v*) in milli-Q water and with respect to the desired resatorvid concentration (the concentration of the gel base must be kept at 20% (*w*/*v*) for the gel to form at room temperature; otherwise, it will remain in a liquid state). Resatorvid was dissolved in PEG-400 using a Branson 2800 sonicator. This formulation was not compounded but mixed with the liquid Pluronic and allowed to solidify prior to being thoroughly mixed to achieve a homogenous gel.

### 2.5. High-Performance Liquid Chromatography (HPLC) Analysis

The HPLC consisted of a Shimadzu LC-2010A HT liquid chromatograph (Torrance, CA, USA), coupled with a UV–Vis dual wavelength detector. The analysis was performed by a reverse-phase HPLC assay, using a Luna C_18_, 5 μm column (250 mm × 2 mm) (Phenomenex, Torrance, CA, USA), maintained at 25 °C ± 2 °C. Ultraviolet detection was done at 254 nm. The mobile phase conditions were 50:50 (*v/v*) acetonitrile: trifluoroacetic acid (0.1% *v/v*) at a flow rate of 0.3 mL/min. The injection volume was 10 µL. The retention time for resatorvid was ~15.9 min. Quantification was determined using peak area and calculated from a five-point standard curve (0.0625 mg/mL to 1 mg/mL, R^2^ = 0.9971). Standards were prepared by volumetric dilution in acetonitrile and stored at 4 °C, protected from light. The modifications made to the method were using a column length of 250 mm × 2 mm instead of 150 mm × 2.1 mm and a mobile phase of 50:50 (*v/v*) acetonitrile: trifluoroacetic acid (0.1% *v/v*) instead of 40:60 (*v/v*) acetonitrile: trifluoroacetic acid (0.1% *v/v*) as previously reported [17]. 

### 2.6. In Vitro Oil–Water Partitioning Coefficient (Log-P) Analysis of Resatorvid 

Resatorvid raw powder was weighed out (0.003 g) and placed in 2 dram amber-colored glass vials containing equal volumes (1.5 mL) of 1-octanol and PBS (1×, pH = 7.4) to make a 1 mg/mL solution. Various solutions were made, and the pH was adjusted and maintained at pH values of 6.5, 7.1, 8.8. Adjustments to the pH were made using 0.1 M hydrochloric acid (HCl) solution and 0.1 M sodium hydroxide (NaOH) solution. The temperatures used in the analysis were 37 °C and 35 °C. The vials were rotated using a Barnstead/Thermolyne Labquake shaker (Dubuque, IA, USA) for 24 h and left undisturbed in a vertical position for an additional 24 h for phase separation between the aqueous and non-aqueous layers. Then, 200 μL of the organic and aqueous layers were sampled very carefully without disturbing the separation of phases and analyzed via the HPLC method previously mentioned.

### 2.7. In Vitro Permeation of Resatorvid through Strat-M^®^ Transdermal Diffusion Membrane

The permeability of resatorvid was determined using Strat-M^®^ transdermal diffusion membranes on a Franz cell apparatus. The receptor compartment was 5 mL, and the effective diffusion area was 0.64cm^2^. The resatorvid formulations were prepared as outlined in the preparation of topical formulations by non-sterile compounding section, under the methods section. A mixture of HPLC-grade ethanol (10% *v/v*) and phosphate-buffered saline (PBS) at pH 7.4 (1×) was used as the receptor medium, which was maintained at 35 °C ± 0.05 °C using a precision reciprocal shaking bath model 25 (Thermo Fisher Scientific, Fair Lawn, NJ, USA) and oscillated at a constant speed of 30 oscillations per minute during the experiment. At predetermined time intervals, 200 µL of the receptor medium was removed and replaced with an equal volume of fresh medium. The flux, at steady state (J), was estimated as the slope of the linear regression analysis of the linear portion of the permeation curve. Lag time (Lt) was defined as the time intercept of the steady-state region of the permeation curve (i.e., x-intercept) [18]. The cumulative amounts of resatorvid that permeated through the Strat-M^®^ membrane were plotted as a function of time and used to determine the previously mentioned permeation parameters. The cumulative amounts of resatorvid that permeated through the membrane and the amount of resatorvid left on the Strat-M^®^ membrane were extracted and quantified via the HPLC method previously mentioned. Drug extraction was quantified following our previously described protocol [17]. Briefly, the Strat-M membrane was removed from the Franz cell, placed in isopropyl alcohol, and sonicated for 10 min using a probe sonicator, followed by centrifugation (1400 rpm, room temperature). After supernatant filtration, a quantitative high-performance liquid chromatography (HPLC) analysis was performed. 

### 2.8. In Vitro Permeation of Resatorvid Using 3D Normal Human-Derived Epidermal Keratinocytes (EpiDerm^TM^)

After receiving EpiDerm^TM^ 3D normal human-derived epidermal keratinocytes and following MatTek’s protocol, the tissue culture inserts were transferred to a 6-well cell culture plate. The culture plate would serve as the receptor compartment and was prefilled with 1 mL of PBS without calcium chloride (CaCl_2_) and magnesium chloride (MgCl_2_) (MatTek, Ashland, MA, USA). The plate was then placed inside a precision reciprocal shaking bath model 25 (Thermo Fisher Scientific, Fair Lawn, NJ, USA) with an oscillation speed of 30 oscillations per minute and at 37 °C ± 0.05 °C. This setup is to mimic that of a Franz cell diffusion cell, where there is a donor/receiver interface. The effective diffusion area was 0.256 cm^2^. The same methodology used in the previously mentioned permeation study using Strat-M^®^ was used for this analysis.

### 2.9. In Vitro Cell Dose–Response Assay in 2D Cell Culture

The effects of raw resatorvid on the viability of human representative keratinocyte cell lines exposed to concentrations at 1000 μM, 100 μM, 10 μM, 1 µM, 0.1 µM, and 0.01 µM were tested. HaCaT cells (a human transformed keratinocyte from a histologically normal skin cell line) and primary NHEKs (normal human epidermal keratinocyte cells) were used as models of the human epidermis, which generates keratinocytes in the stratum basale and then differentiates into the final barrier layer of the skin, the stratum corneum. The HaCaT cells were grown in Dulbecco’s modified Eagle’s medium (DMEM), Optimized 1×, 10% (*v/v*) FBS, and Pen-Strep (100 units/mL penicillin, 100 µg/mL) in a humidified incubator at 37 °C and 5% CO_2_. The NHEK cells were grown in an NHEK growth medium provided by MatTek (Ashland, MA, USA) in a humidified incubator at 37 °C and 5% CO_2_. 

After confluence, the HaCaT and NHEK cells were seeded in a black 96-well plate at a concentration of 5000 cells in 100 µL of media/well, then incubated for 48 h to allow attachment to the surface of the plates. After 48 h, the cells were then exposed to the different concentrations of resatorvid previously mentioned. The drug solutions were prepared by dissolving raw resatorvid powder in 5% (*v/v*) DMSO and adequate growth medium to make a solution at 1×. A volume of 100 µL of drug solution was added to each well. After 48 h of exposure and incubation at 37 °C and 5% CO_2_, 20 µL of 20 µM resazurin sodium salt were added to each well and incubated for 4 h. The fluorescence intensity of resorufin was detected at 544 nm (excitation) and 590 nm (emission) using the Molecular Devices^®^ SpectraMax^®^ M3 Multi-Mode Microplate Reader (Sunnyvale, CA, USA). The relative viability of the cells was calculated as followed by Equation (1):

(1) $$Relative\;Viability\;\%=\frac{Sample\;flourescence\;intensity}{Control\;flourescece\;intensity}\times 100\%\;$$

### 2.10. Statistical Analysis 

The data are presented as the mean ± standard deviation, derived from three independent experiments. The statistical analysis among groups was performed using a one-way analysis of variance (ANOVA) for resatorvid diffusion through Strat-M membrane. A value of *p* < 0.05 was considered as significant.

## 3. Results

### 3.1. Physicochemical Characterization of Raw Resatorvid

The SEM micrographs (Figure S1) of raw resatorvid showed irregularly shaped particles of varying size. Resatorvid had characteristics of a crystal with a shape ranging from square to needle-like. The average geometric (linear) size of the raw particles was 5.84 µm (range: 0.024 µm to 349 µm). The EDX spectrum of the raw drug is shown in Figure S2. For chemical identification, the characteristic Kα line (peak) of chlorine (Cl) is seen at 2.6 keV; the Kα line of fluorine (F) is seen at 0.68 keV; and the Kα line of sulfur (S) is seen at 2.3 keV. The Kα line of carbon (C) is seen at 0.3 keV, and the Kα of oxygen (O) is seen at 0.5 keV. The peaks corresponding to S, Cl, and F are representative of resatorvid. The XRPD spectrum of the raw drug (Figure S3) showed intense diffraction peaks at 13.66°, 19.65°, 25.2°, 27.2°, and 30.78° (2θ), indicating the crystalline nature of the drug. The DSC thermogram of raw resatorvid (Figure S4) exhibited two endothermic transitions at ~ 53 °C and 69 °C. The transition at 53°C corresponds to a solid–solid phase transition, and the other transition at 69 °C corresponds to the melting of the drug. The enthalpy and temperature values are summarized in Table S1. The predicted melting point of resatorvid is ~69 °C. 

Figure S5 shows the HSM images of the raw drug at various temperature points. Raw resatorvid showed irregularly shaped particles with birefringence, indicating the crystalline nature of the drug. At 50–52 °C, the particles showed a glass phase transition, followed by melting at ~71 °C with droplet formation and disappearance of birefringence. The residual water content was quantified by Karl Fisher coulometric titration. As seen in Table S2, raw TAK-242 had an average water content of 0.071 % (*w*/*v*) ± 0.016. Characteristic Raman bands appeared in different wavelengths of the spectrum (Figure S6). The most representative for resatorvid were at 878 cm^−1^ (C–Cl antisymmetric stretch), at 1649 cm^−1^ (Ar–NH bend), at 1235 cm^−1^ (COC stretching), and at 1269 cm^−1^ (Aromatic C–H bend) as previously reported [19,20]. Figure S7 shows the ATR-FTIR spectrum of raw resatorvid; at approximately 1492 cm^−1^ (SO_2_ antisymmetric stretching), characteristics of a sulfonamide group appeared. The peaks at a range of 1244 cm^−1^ to 1149 cm^−1^ indicate that a C-F stretch is observed. The peak at 1706 cm^−1^ (C = O stretch) is indicative of an α-ß unsaturated ketone as previously reported [20,21]. The spectral pattern seen at the fingerprint region (<2000 cm^−1^) was consistently observed in raw resatorvid.

### 3.2. Preformulation of Resatorvid: Solubility and Compatibility

Different solvents and volumes were used for each of the bases and were optimized to find a compatible solvent and volume, to the point where the base would not lose its consistency and become liquid-like. PENcream^TM^ (cream formulation) was compatible at all concentrations (5%, 2.5%, and 1.25%) with PEG-600 and a total volume of 0.5 mL for 1 g of base. DermaBase^TM^ (cream formulation) was compatible at all concentrations using PEG-600 and a volume of 1.0 mL for 1 g of base; the lotion formulation was compatible with PG as the solvent and a volume of 1.0 mL for 1 g of base. The carbomer gel formulation and hyaluronic acid formulation were both compatible at 2.5% and 1.25% concentrations of the drug with PEG-400 as the solvent with volumes of 1.0 mL and 0.5 mL, respectively, for 1 g of base. The poloxamer formulation was compatible with PEG-400 at 2.5% and 1.25% concentrations of the drug. The volume of the solvent in the poloxamer will vary depending on quantity, since the poloxamer base has to be at 20% (*w*/*v*). 

### 3.3. In Vitro Oil–Water Partitioning Coefficient (Log-P) Analysis of Resatorvid 

The predicted pKa of resatorvid was 6.58 using Molecular Operating Environment (MOE^®^) software (Montreal, Canada), and the experimentally determined pKa reported was 8.0–8.1. The predicted Log-P (cLogP) of TAK-242 was 2.53 using ChemDraw™ Ver. 16.0 (Cambridge Soft, Cambridge, MA, USA), and 3.04 using Swiss ADME (Swiss Institute of Bioinformatics, Switzerland). The partition coefficient of resatorvid was determined using three different pKa’s (6.5, 7.1, 8.8) based on no primary literature determining the nature of it. As shown in Table 1**,** the partition coefficient (Log-P) of resatorvid at 35 °C is within a range of 0.94–1.68, and at room/ambient temperature, is within a range of 0.88–1.58.

### 3.4. High-Performance Liquid Chromatography (HPLC) Analysis

**Figure 2:**
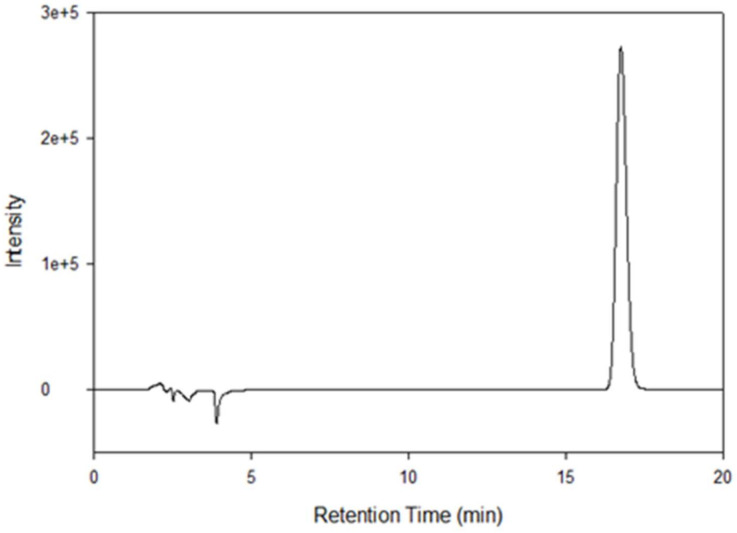
HPLC chromatogram of resatorvid. Format like 1e+5 means 1 ×10^5^.

The high-performance liquid chromatography (HPLC) analysis showed that resatorvid had an average retention time of 15.9 ± 0.01 min, as shown in Figure 2.

### 3.5. In Vitro Permeation of Resatorvid (TAK-242) through Strat-M^®^ Transdermal Diffusion Membrane

**Figure 3:**
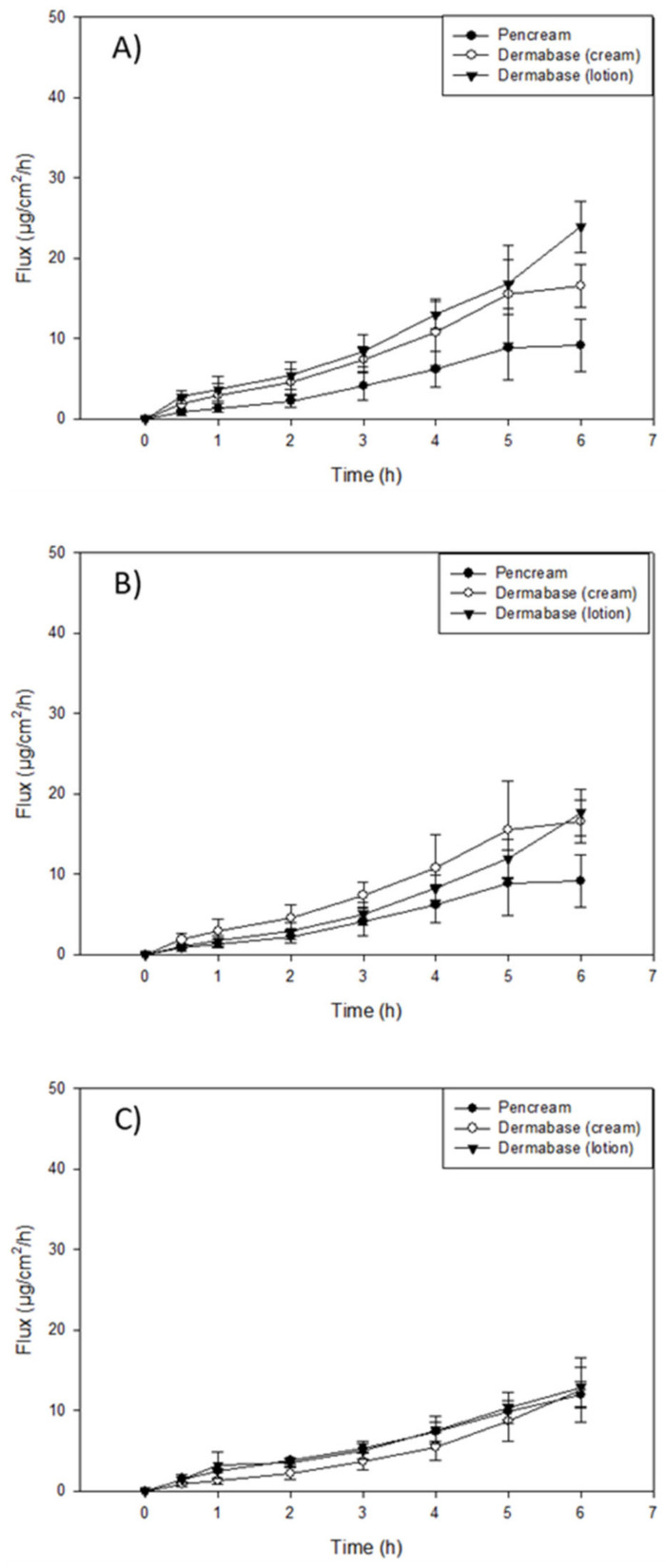
In vitro Franz cell/Strat-M^®^ permeation profile of resatorvid: (**A**) 5% creams and lotion formulations; (**B**) 2.5% creams and lotion formulations; and (**C**) 1.25% creams and lotion formulations.

**Figure 4:**
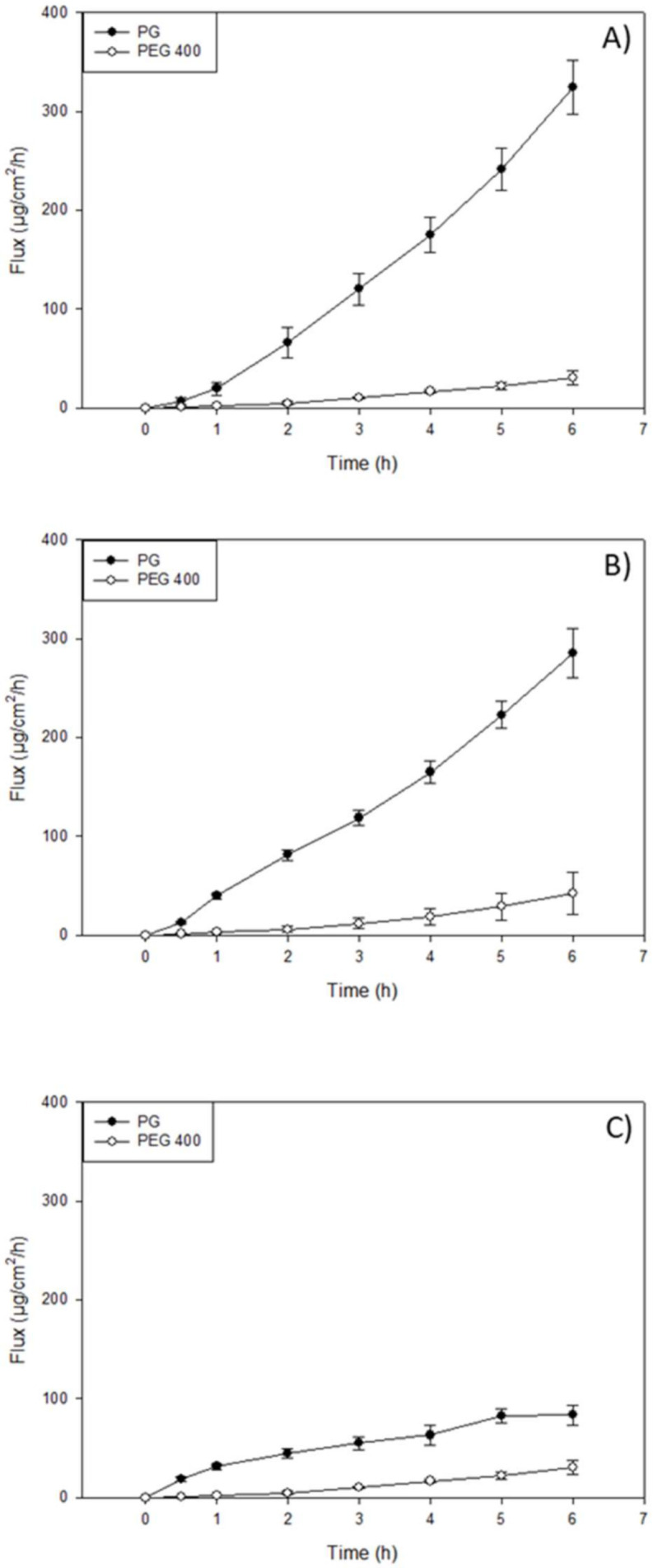
In vitro Franz cell/Strat-M^®^ permeation profile of resatorvid: (**A**) 5% simple solutions; (**B**) 2.5% simple solutions; and (**C**) 1.25% simple solutions.

**Figure 5:**
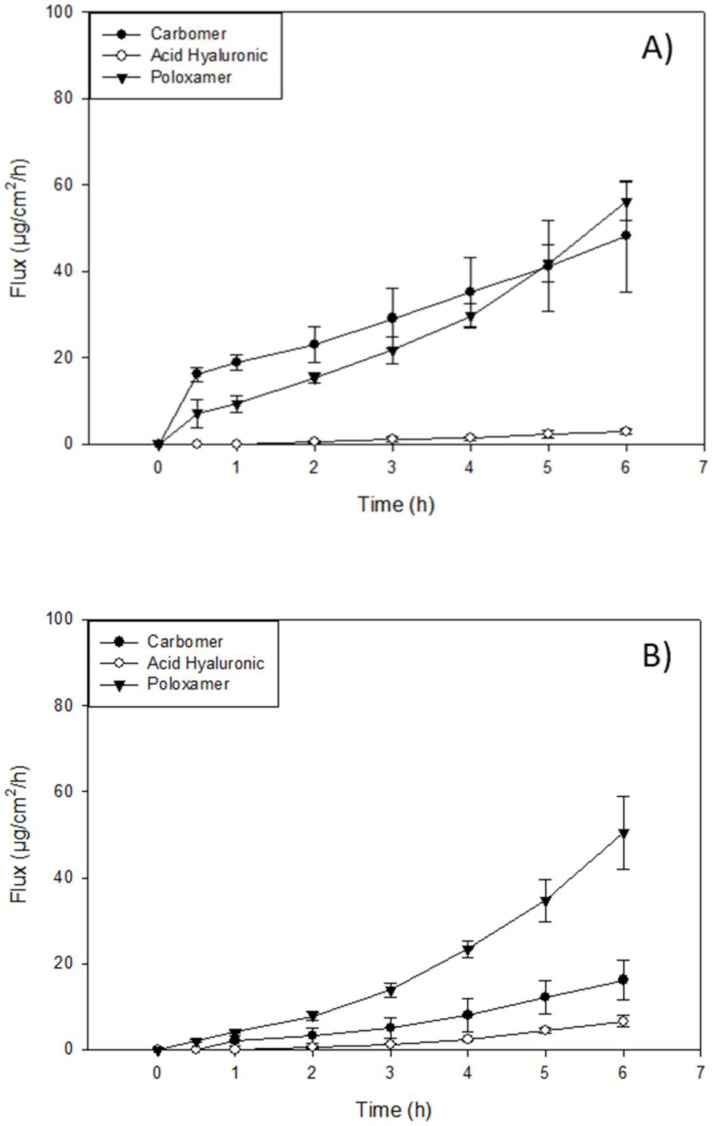
In vitro Franz cell/Strat-M^®^ permeation profile of resatorvid: (**A**) 2.5% gel formulations and (**B**) 1.25% gel formulations.

In vitro transdermal diffusion and permeation evaluation were performed using a Strat-M^®^ transdermal diffusion membrane as a synthetic non-animal-based model for transdermal diffusion in human skin. The analysis was conducted on the formulations of TAK-242 at 1.25%, 2.5%, and 5%, listed under the methods section on the preparation of topical formulations by non-sterile compounding. Figure 3, Figure 4 and Figure 5 illustrate flux versus time from the resatorvid formulations. The drug managed to diffuse through the membrane in all formulations, although a higher drug amount was found in the membrane. Among the six formulations, 1.25% (*w*/*w*) carbomer and both hyaluronic acid HD (1.25% and 2.5%) were exhibiting lag times, which was calculated from the linear equation (F(x) = 0) at the steady state of the drug’s permeated profile. The 1.25% and 2.5% (*w*/*w*) hyaluronic acid HD showed lag times of 1.98 ± 0.34 and 1.14 ± 0.25 h, respectively, and carbomer showed a very short lag time of 0.66 ± 0.17 h. Moreover, the PEG-400 solutions exhibited lag times of 1.14 ± 0.34 h at 1.25% (*w*/*v*), 0.83 ± 0.15 h at 2.5% (*w*/*v*), and 2.4 ± 0.29 h at 5% (*w*/*v*). The Strat-M^®^ permeation flux (µg/cm^2^/h), lag time (h), and drug retention in the membrane (µg) parameters are shown in Table 2, Table 3 and Table 4. 

The highest mass flux (J) values found in oil-based formulations were for the DermaBase^TM^ (cream) at all concentrations tested, with 8.72 ± 2.59, 3.70 ± 0.64, and 2.82 ± 0.69 for 5, 2.5, and 1.25% (*w*/*w*), respectively (Table 2). DermaBase^TM^ formulations showed a significant difference compared to PENcream^TM^ only at 5% (*w*/*w*) (*p* < 0.05). On the other hand, for HD formulations, Pluronic^®^ F-127 showed the higher J in both concentrations tested. The in vitro studies exhibited statistically significant increased (*p* < 0.05) diffusion of resatorvid from the carbomer and Pluronic^®^ F-127 HD when compared with the hyaluronic acid at 2.5% (*w*/*w*). For 1.25% (*w*/*w*) HD, Pluronic^®^ F-127 exhibited a higher permeation (µg/cm^2^/h) rate (*p* < 0.05) compared to the other two HD. This may be indicative of a drug concentration effect on the permeation profile of the formulations for certain ones, as it seems to increase proportionally with an increase in drug concentration (*p* < 0.05), as with DermaBase^TM^ (cream) in Table 2. Additionally, for resatorvid in solution, it can be observed that the type of solvent used significantly affects the permeation, allowing the discrimination between the effect of the solvent and the type of vehicle used. This behavior can be explained due to the hydrophobic character of resatorvid (log P = 1.68). The hydrophilic HD allowed greater permeation and a higher flow of resatorvid in a shorter time. For the oil-based creams, a decrease in these parameters was observed due to a greater affinity between the hydrophobic drug and the formulation [22].

On the other hand, among oil-based creams, DermaBase^TM^ (Lotion) at 5% (*w*/*w*) showed the highest resatorvid retention after the permeation test, with 570.7 ± 55.87 μg (*p* < 0.05), followed by DermaBase^TM^ (cream) and PENcream^TM^ showing values of 409.24 ± 12.67 μg and 397.63 ± 16.22 μg, respectively. For 2.5% (*w*/*w*) HD, all formulations showed statistical difference (*p* < 0.05) for drug retention, with carbomer having the higher resatorvid retention of 638.51 ± 32.19 µg. For lower concentrations tested, 1.25% (*w*/*w*) carbomer and hyaluronic acid HD were different (*p* ≥ 0.05), with 638.51 ± 32.19 µg and 415.36 ± 21.19 µg, from Pluronic^®^ F-127 (poloxamer triblock copolymer) (Table 4). These are valuable results for the qualification of semisolid dosage forms in the preformulation stages, since these values help to predict the behavior of topical dosage forms.

### 3.6. In Vitro Cell Dose–Response Assay in 2D Cell Culture

**Figure 6:**
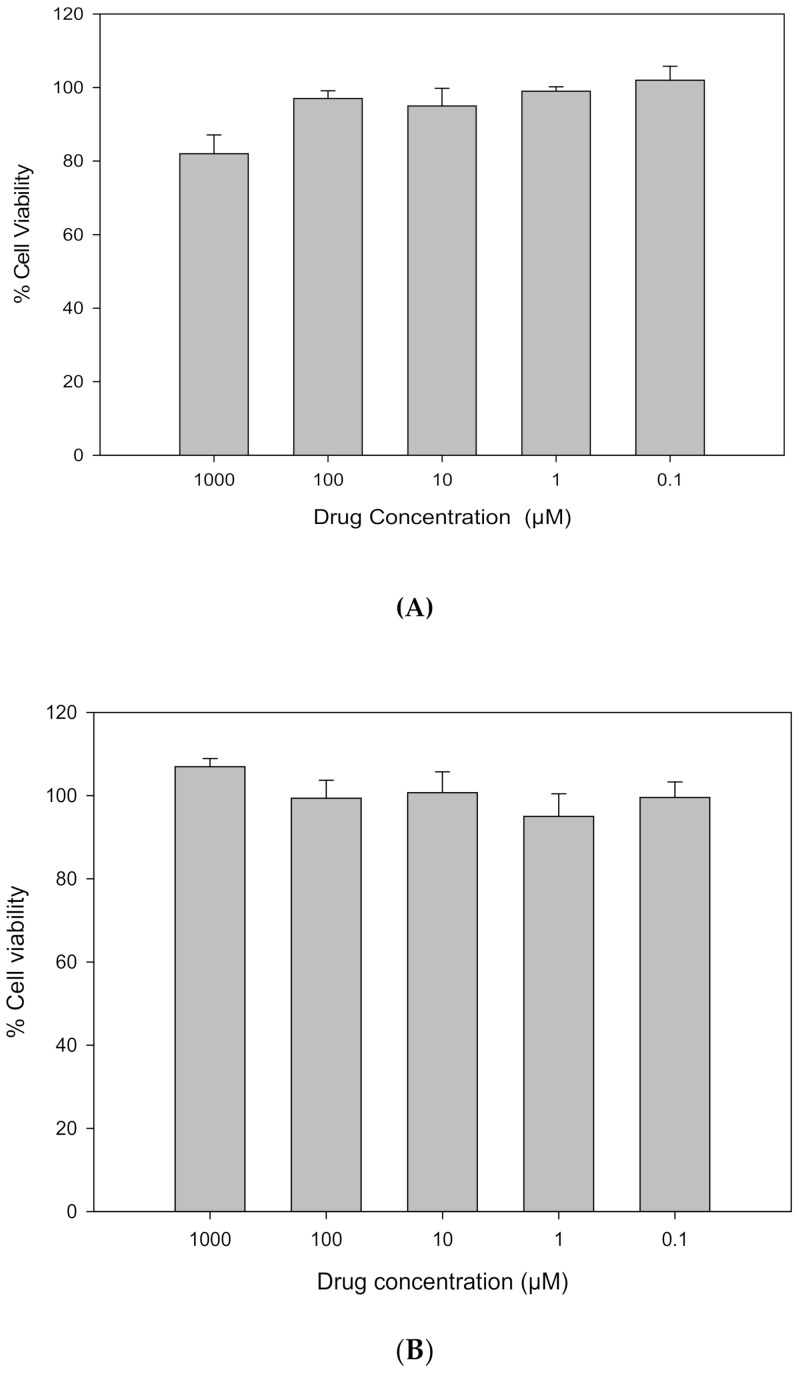
In vitro cell viability of raw resatorvid on (**A**) human transformed keratinocytes (HaCaT) and (**B**) primary normal human epidermal keratinocytes (NHEKs).

Resatorvid did not show any significant toxicity in either human transformed keratinocytes (HaCaT), as shown in Figure 6A, or primary normal human epidermal keratinocytes (NHEKs), as shown in Figure 6B, after 72 h exposure to a series of increasing concentrations of raw resatorvid. The viability of NHEK cells remained nearly 100% at all concentrations; the viability of HaCaT cells was close to 100% for concentrations at 0.01 µM and 0.1 µM, and as the concentration increased from 1 µM to 1000 µM, cell proliferation was observed. 

### 3.7. In Vitro Permeation of Resatorvid (TAK-242) through 3D Normal Human-Derived Epidermal Keratinocytes (EpiDerm^TM^)

**Figure 7:**
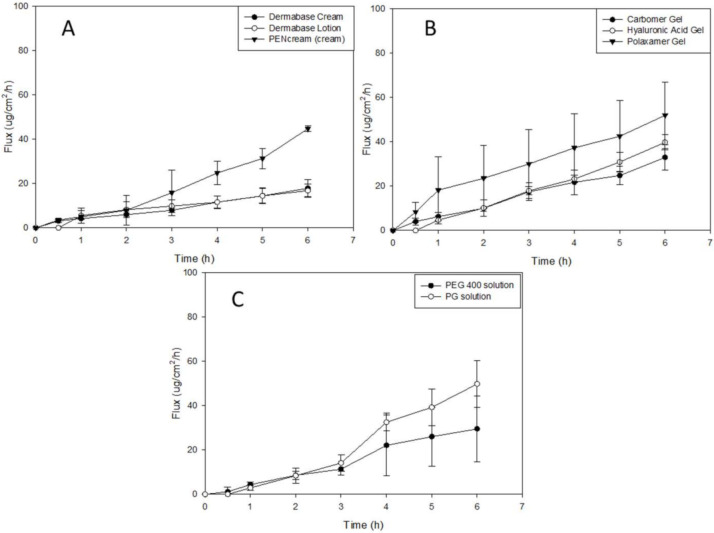
In vitro EpiDerm^TM^ permeation profile of resatorvid: (**A**) 1.25% cream formulations; (**B**) 1.25% hydrogels formulations; and (**C**) 1.25% solutions.

Drug permeation studies were also performed using Epiderm™ (3D normal human-derived epidermal keratinocytes) for formulations at a concentration of 1.25%. Sampling was done over the course of 6 h, as shown in Figure 7. The flux values from 1.25% (*w*/*w*) DermaBase^TM^ formulations were the highest, followed by 1.25% (*w*/*w*) hyaluronic acid, with 7.90 ± 1.90 µg/cm^2^/h and 6.53 ± 0.769 µg/cm^2^/h, respectively. From the eight formulations, the DermaBase^TM^ lotion formulation, PEG-400 and PG solutions, and hyaluronic acid HD showed lag times ≤ 0.5 h. For drug retention, carbomer gel formulation showed a higher value than the resatorvid solutions: 51.06 ± 12.4 µg, 43.07 ± 6.91 µg, and 21.35 ± 6.54 µg, respectively. For oil-base creams, PENcream^TM^ showed the highest drug retention with 19.69 ± 2.16 µg, followed by DermaBase^TM^ cream with 14.24 ± 2.54 µg. Diffusion through the 3D cell model is relevant, as it will help predict resatorvid skin-depth penetration capacity and drug accumulation in the tissue, all of which are essential for topical delivery of high-molecular-weight and hydrophobic molecules into skin at a high risk for cancer development.

## 4. Discussion

Resatorvid is a small molecular TLR4 antagonist with proven activity to prevent UV-induced nonmelanoma skin cancer when used topically on animal models [7,17,23]. Despite its pharmacological activities, TAK-242 presents physicochemical limitations, such as low aqueous solubility and bioavailability, reducing its permeation across the skin. The clinical efficacy of bioactive molecules administered by topical route depends mainly on their physicochemical and pharmacological properties, as well as their bioavailability at the site of action, which is limited by the low permeability of the stratum corneum. Therefore, due to the special structure and properties of the skin, novel formulations, such as hydrogels and oil-based creams, have been developed in an attempt to overcome those limitations and, in addition, to increase residence time on the skin. The beneficial therapeutic effect of TAK-242 has been tested, not only for cancer prevention by binding to toll-like receptors, but also for its demonstrated inhibition of serum cytokine levels and improved survival in a mouse sepsis model when it was co-administered with antibiotics after intraperitoneal injection [24]. In addition, TAK-242 has the potential to be developed as a therapeutic agent against rheumatoid arthritis, showing no adverse effects with the systematic administration of resatorvid [25].

Therefore, a topical resatorvid formulation is needed for further preclinical and clinical studies. In this study, we have successfully created and developed eight topical formulations of TAK-242 with FDA-approved excipients that are commonly used in the pharmaceutical and cosmetic industries, including solutions (PG and PEG-400), hydrogels (carbomer, Pluronic^®^, and HA), and creams and lotions (PENcream^TM^ and DermaBase^TM^). A physicochemical characterization of resatorvid was completed. The transitions observed during HSM (Figure S5) were consistent with DSC thermograms (Figure S4). Biocompatibility and cell viability evaluation on the human skin cell lines, HaCaT and NHEK, demonstrated that resatorvid had negligible toxicity in both cell lines, suggesting a good safety profile over a wide drug dose range. 

The skin is the largest and most readily accessible organ, and a significant barrier for both transdermal and topical drug delivery. The stratum corneum (SC) is the outermost layer of human skin, which is considered the major resistance to transdermal transport. The SC has a multilayer structure, and each layer consists of an intercellular lipid matrix filled with corneocytes in a brick-and-mortar arrangement [26]. Due to the complexity of the structure and chemical compositions of the human epidermis, it is very critical to find a reproducible and convenient in vitro model to predict drug permeation through the skin in the early stage of formulation development. The Strat-M^®^ membrane is a synthetic model designed to mimic the structure and lipid composition of human skin. Each Strat-M^®^ membrane has a top layer that resembles the SC, and two layers of porous polyether sulfone mimicking the skin dermis, on top of one layer of polyolefin non-woven fabric support [27]. In addition, the Strat-M^®^ membrane is filled with a blend of lipids that are similar to those found in the human skin [27]. Due to its cost-efficiency and reproducibility, the Strat-M^®^ membrane has been widely used to evaluate topical formulations [27,28,29,30,31]. 

Penetration enhancers are usually surfactants that have the potential to alter the lipid integrity within the stratum corneum, and their choice in formulation development is based on drug efficacy and their own effects on the skin as well. Bile acids have been extensively investigated for their drug transport enhancement properties across different biological membranes, following the specific routes of drug administration [32]. In the drug permeation study using a Strat-M^®^ membrane, the highest drug retention in the membrane was found in the PG solution groups at three drug concentrations (Table 3). Similarly, the PG solution showed the highest flux through the membrane at three drug concentrations compared to the other formulations (Table 3). DermaBase^TM^ cream showed a higher drug flux and higher drug retention compared to DermaBase^TM^ lotion and PENcream^TM^ (*p* < 0.05) (Table 2). The ability of TAK-242 to penetrate into and through the Strat-M^®^ membrane demonstrates its potential for treating or preventing NMSCs in the clinic. TAK-242 retained in the membrane mimics the skin retention, where the activation of TLR4 may occur. All these results showed that the formulations allowed minimal permeation of the drug through the skin, thereby ensuring a possible reduction in the systemic toxicity and more accumulation in the target site.

PENcream™ and DermaBase™ are elegant, oil-in-water vanishing creams formulated for enhanced transdermal penetration when you want to incorporate both water- and oil-soluble ingredients and a more efficacious active drug absorption. Both ointments, PENcream™ and DermaBase™, showed a correlation of higher flux and drug retention while increasing TAK-242 in the formulations. This result suggests that modifying the formulation concentrations can influence the change in permeability. The degree of saturation can be increased by increasing the medication concentration in the vehicle. It is especially desirable to create a supersaturated drug solution in the vehicle, since this will induce a thermodynamic drive for the drug to diffuse out of the vehicle and into the skin [33]. Lu, Lou [34] produced a topical application of caffeine and derivatives with DermaBase™, showing that applications of caffeine derivatives to UVB-pretreated ‘high-risk mice’ inhibits skin carcinogenesis (keratoacanthomas and squamous cell carcinomas) and tumor growth.

Several studies have reported regarding the development of a semisolid skin delivery system with different types of Pluronic^®^, which has been widely proposed for this purpose. Vigato, Querobino [35] postulated that the permeation of hydrophobic drug amounts with a Pluronic^®^ system can be attributed to some structural factors, such as the hydrophobic interaction of the drug, and the oil-phase promoting drug solubilization and acting as a permeation enhancer. In addition, Pluronic^®^ was associated with the hydrophobic drug interacting with its polyoxypropylene (poly(propylene oxide)) hydrophobic unit on the organic–aqueous phase interface and also with promoting the drug into the Strat-M lipid matrix. Strat-M^®^ has been employed as a skin model for evaluating drug diffusion profiles from innovative delivery systems throughout the early stages of research, despite the discrepancies between in vitro (using artificial membranes) and ex vivo skin permeation profiles. In fact, the lipid matrix composition (ceramides, free fatty acids, cholesterol, and phospholipids) can mimic the skin barrier, making it useful for assessing penetration parameters for various pharmaceutical formulations, such hydrogels and oil-base creams [29].

For the carbomer formulation, hydrogen ions (H^+^) are dissociated from the carboxyl groups of polymer when the solution is neutralized with a basic chemical (e.g., triethanolamine employed in this study), and the charge of the polymer chains is changed to negative. The anionic polymer chains can then become maximally uncoiled due to electrostatic repulsion, resulting in increased viscosity and swelling, a process known as gelation. Interestingly, Kim, Kim [36] used a hydrogel base on carbomer for 20(S)-protopanaxadiol (PPD) for the study of epidermal/dermal deposition. In their study, they used Strat-M, observing that the deposited amount of 20S-PPD was significantly higher in F5-H than in the two control groups; they attributed their observations of the increase in the partition coefficient of the drug between the formulation and skin to the permeation enhancers used, and to the hydration of the stratum corneum by the carbomer, reducing the barrier function and enhancing drug diffusion through the skin [37]. 

For several years, HA-based hydrogels have been a hot topic in the treatment of skin diseases, and are a promising option for dermal drug delivery. It has been shown that the enhancement effect of drug absorption using HA hydrogels for dermal administration is most likely due to a combination of skin hydration, interaction with keratin structure, and protein-HA cotransport, which aids transport across the stratum corneum as well as HA absorption [38]. In our study, carbomer and HA-based hydrogel showed similar drug retention in the membrane: 445 vs. 415 µg, respectively. This may be due to the same hydration process of the skin-enhancing penetration to the epidermis layer.

Comparing the 2.5% *w*/*w* hydrogel formulations, there are notable differences in flux and drug retention in the carbomer and hyaluronic acid formulations. Carbomer showed a higher drug retention and flux when compared to poloxamer and hyaluronic acid formulations, but hyaluronic acid showed minimum flux and intermediate drug retention. This can be attributed to the base, as these agents are hydrophilic thickening agents that may not be able to fully penetrate the skin and may allow for TAK-242 to be retained. The other component is the vehicle in which the drug is dissolved, in this case PEG-400, which may contribute to the difference in flux. Another aspect to consider in the gels is the molecular weight of the bases, as that will impact the permeation of the drug onto the skin, which can be further illustrated by the data presented [39].

Another in vitro model employed in this study was the EpiDerm^TM^ model, which was a highly differentiated, three-dimensional tissue model consisting of multiple layers of normal human-derived epidermal keratinocytes (NHEKs) on cell culture inserts [40]. The EpiDerm^TM^ model exhibits in vivo like morphology, lipid profile, and metabolic activity, which reproduces some of the barrier functions of normal human skin; hence, it has been used for assessing skin irritancy, corrosivity, phototoxicity, and drug transport as well [41]. In the permeation study incorporating EpiDerm^TM^, the PG solution exhibited higher drug flux and permeability in the EpiDerm^TM^ compared to other formulations (Table 5). PENcream^TM^ lotion outperformed other creams and lotions in drug flux and permeability. Although carbomer gel exhibited a comparable flux and permeability relative to HA and Pluronic^®^ gels, higher drug retention was found in carbomer-gel-treated EpiDerm^TM^ in comparison to PG solution and other gel formulations. (Table 5).

Strat-M was used as an early screening model to select the best performing formulation concentration and platform, based on membrane drug retention and permeation, to be tested in the EpiDerm™ transdermal permeation study. EpiDerm^®^ is a highly differentiated 3D tissue model consisting of normal human-derived epidermal keratinocytes (NHEKs). It is not intended to simulate all the skin layers, and it only simulates the epidermis, which includes 8 to 12 cell layers. While there cannot be a direct comparison between synthetic membranes, some flux values can correlate well using both models, with the exception of drug retention, as it is much higher in the Strat-M due to the different layers that compose it; therefore, more drugs may be retained.

While differences in the permeation, retention, and drug flux can be attributed to the different characteristics of the Strat-M^®^ membrane and the EpiDerm^TM^ membrane, it can also be attributed to the components of each formulation. The formulations, as previously described, were composed of a cream, lotion, and hydrogel, which all had TAK-242 dissolved in either PEG or PG and then incorporated in the formulation. Given that PEG and PG are considered penetration enhancers and may increase permeation by promoting diffusion, partitioning, or drug solubility of an active ingredient through the stratum corneum or, in this case, our membranes, it may influence the permeation, retention, and drug flux of resatorvid [42]. Furthermore, PENcream^TM^ and DermaBase^TM^ are specifically designed to promote dispersion and dissolution of TAK-242, but may also promote penetration into the skin layers and, therefore, affect the parameters previously mentioned. 

## 5. Conclusions

This systematic and comprehensive study reports for the first time on eight innovative topical formulation platforms that were successfully created, along with in vitro drug membrane permeation on in vitro models (synthetic Strat-M membrane and human 3D EpiDerm skin). In addition, the physicochemical properties of the resatorvid drug were reported for the first time. Moreover, in vitro cell viability was successfully conducted on two human skin cell types (HaCaT skin cell line and primary NHEK skin cells), demonstrating biocompatibility as a function of drug dose. In vitro drug permeation studies were utilized to screen the superior formulation candidates from different categories (solution, gel, and cream/lotion). PG solution, carbomer gel and DermaBase^TM^ cream demonstrated better drug retention in in vitro models.

## Figures and Tables

**Table 1:** Summary of partition coefficient (Log-P) for resatorvid at various pH (n = 3, mean ± standard deviation).

Experimental (Log-P)	Average ± SD
At 35 °C, pH = 6.5	1.65 ± 0.029
At Room temperature/ambient temperature, pH = 6.5	1.58 ± 0.019
Experimental (Log-P)	Avg. Log-P ± SD
At 35 °C, pH = 7.1	1.68 ± 0.013
At Room temperature/ambient temperature, pH = 7.1	1.54 ± 0.149
Experimental (Log-P)	Avg. Log-P ± SD
At 35 °C, pH = 8.8	0.94 ± 0.023
At Room temperature/ambient temperature, pH = 8.8	0.88 ± 0.060
Predicted (cLogP)	Predicted value
ChemDraw Version 16.0	2.53
Swiss ADME	3.04

**Table 2:** In vitro drug membrane parameters of resatorvid topical creams and lotion formulations through Strat-M^®^ transdermal diffusion membrane (*n* = 3, mean ± standard deviation). Lowercase letters indicate differences between formulations with the same concentration. Capital letters indicate difference between concentrations in the same formulation (*p* < 0.05).

5% Creams and Lotions	Flux (µg/cm^2^/h)	Lag Time (h)	Drug Retention (µg)
5% (*w*/*w*) DermaBase^TM^ (cream formulation)	8.72 ± 2.59 a A	-	409.24 ± 12.67 a A
5% (*w*/*w*) DermaBase^TM^ (lotion formulation)	6.03 ± 2.01 a A	-	570.7 ± 55.87 b A
5% (*w*/*w*) PENcream^TM^ (cream formulation)	1.68 ± 0.30 b A	-	397.63 ± 16.22 a A
2.5% creams and lotions	Flux (µg/cm^2^/h)	Lag time (h)	Drug retention (µg)
2.5% (*w*/*w*) DermaBase (cream formulation)	3.70 ± 0.64 a B	-	376.03 ± 50.36 ab A
2.5% (*w*/*w*) DermaBase^TM^ (lotion formulation)	2.85 ± 0.37 a B	-	417.97 ± 24.26 b B
2.5% (*w*/*w*) PENcream^TM^ (cream formulation)	1.66 ± 0.65 a A	-	326.89 ± 24.56 a A
1.25% creams and lotions	Flux (µg/cm^2^/h)	Lag time (h)	Drug retention (µg)
1.25% (*w*/*w*) DermaBase^TM^ (cream formulation)	2.82 ± 0.69 a B	-	199.43 ± 16.63 a B
1.25% (*w*/*w*) DermaBase^TM^ (lotion formulation)	1.98 ± 0.29 a B	-	153.50 ± 32.36 a C
1.25% (*w*/*w*) PENcream^TM^ (cream formulation)	2.03 ± 0.13 a A	-	204.56 ± 12.16 a B

**Table 3:** In vitro drug membrane parameters of resatorvid topical simple solutions through Strat-M^®^ transdermal diffusion membrane (*n* = 3, mean ± standard deviation). Lowercase letters indicate differences between formulations with the same concentration. Capital letters indicate differences between concentrations in the same formulation (*p* < 0.05).

5% Simple Solutions	Flux (µg/cm^2^/h)	Lag Time (h)	Drug Retention (µg)
5% (*w*/*v*) PEG-400 solution	5.33 ± 1.05 a A	2.4 ± 0.29	556.76 ± 47.72 a A
5% (*w*/*v*) PG solution	57.10 ± 3.90 b A	-	1686 ± 285.15 b A
2.5% simple solutions	Flux (µg/cm^2^/h)	Lag time (h)	Drug retention (µg)
2.5% (*w*/*v*) PEG-400 solution	9.16 ± 1.39 a A	0.83 ± 0.15	498.21 ± 37.7 a A
2.5% (*w*/*v*) PG solution	47.87 ± 3.69 b B	-	1242.88 ± 63.76 b B
1.25% simple solutions	Flux (µg/cm^2^/h)	Lag time (h)	Drug retention (µg)
1.25% (*w*/*v*) PEG-400 solution	3.60 ± 1.5 a A	1.14 ± 0.34	264.63 ± 9.47 a A
1.25% (*w*/*v*) PG solution	5.33 ± 1.05 a C	-	938.08 ± 56.73 b B

**Table 4:** In vitro drug membrane parameters of resatorvid topical gels/serum formulations through Strat-M^®^ transdermal diffusion membrane (*n* = 3, mean ± standard deviation). Lowercase letters indicate differences between formulations with the same concentration. Capital letters indicate differences between concentrations in the same formulation (*p* < 0.05).

2.5% Gels and Serum	Flux (µg/cm^2^/h)	Lag Time (h)	Drug Retention (µg)
2.5% (*w*/*w*) Carbomer (gel formulation)	5.76 ± 2.08 a A	-	638.51 ± 32.19 a A
2.5% (*w*/*w*) Hyaluronic Acid (gel/serum formulation)	0.52 ± 0.14 b A	1.14 ± 0.25	472.36 ± 52.29 b A
2.5% (*w*/*v*) Pluronic^®^ F-127 (gel formulation)	8.5 ± 0.39 a A	-	289.72 ± 20.46 c A
1.25% gels and serum	Flux (µg/cm^2^/h)	Lag time (h)	Drug retention (µg)
1.25% (*w*/*w*) Carbomer (gel formulation)	2.77 ± 0.80 a A	0.15 ± 0.07	445.06 ± 85.12 a B
1.25% (*w*/*w*) Hyaluronic Acid (gel/serum formulation)	1.16 ± 0.19 a A	1.98 ± 0.34	415.83 ± 21.19 a A
1.25% (*w*/*v*) Pluronic^®^ F-127 (gel formulation)	8.45 ± 1.51 b A	-	235.06 ± 45.74 c A

**Table 5:** In vitro drug membrane parameters of resatorvid topical formulations through EpiDerm^TM^ -D normal human derived epidermal keratinocytes as models for transdermal diffusion (*n* = 3, mean ± standard deviation).

1.25% Creams and Lotion	Flux (µg/cm^2^/h)	Lag Time (h)	Drug Retention (µg)
1.25% (*w*/*w*) DermaBASE^TM^ (cream formulation)	2.56 ± 0.68	-	14.24 ± 2.54
1.25% (*w*/*w*) DermaBASE^TM^ (lotion formulation)	1.97 ± 0.38	0.20±0.08	9.38 ± 2.56
1.25% (*w*/*w*) PENcream^TM^ (cream formulation)	7.9 ± 1.90	-	19.69 ± 2.16
1.25% simple solutions	Flux (µg/cm^2^/h)	Lag time (h)	Drug retention (µg)
1.25% (*w*/*v*) PEG-400 solution	2.39 ± 0.68	0.37 ± 0.11	21.35 ± 6.54
1.25% (*w*/*v*) PG solution	11.08 ± 2.92	0.50 ± 0.16	43.07 ± 6.91
1.25% gels and serums	Flux (µg/cm^2^/h)	Lag time (h)	Drug retention (µg)
1.25% (*w*/*w*) Carbomer (gel formulation)	5.34 ± 1.27	-	51.06 ± 12.4
1.25% (*w*/*w*) Hyaluronic Acid (gel/serum formulation)	6.53 ± 0.769	0.36 ± 0.0.18	24.02 ± 6.63
1.25% (*w*/*v*) Pluronic^®^ F-127 (gel formulation)	6.37 ± 0.47	-	18.52 ± 2.08

## Data Availability

The data presented in this study are available on request from the Corresponding Author.

## Supplementary Materials

The following are available online at https://www.mdpi.com/article/10.3390/pharmaceutics14040700/s1, Figure S1: SEM micrographs of resatorvid at 400×, 1300×, 3000× and 5000× resolution. Figure S2. EDX spectra of raw resatorvid powder showing characteristic peaks. C-Carbon, O-Oxygen, S-Sulfur, F-Floride, and Cl-Chlorine atoms. Figure S3: XRPD diffraction patterns of raw resatorvid. Figure S4. DSC thermogram of raw resatorvid. Figure S5. Representative HSM images of raw resatorvid. Figure S6. Representative Raman spectra of raw resatorvid using 785 nm laser. Figure S7. ATR-FTIR spectrum of raw resatorvid. Table S1: DSC thermal analysis (*n* = 3, mean ± standard deviation). Table S2. Residual water content quantified by KFT. (*n* = 4, mean ± standard deviation).
