# Supplementary material for: Design, Physicochemical Characterization, and In Vitro Permeation of Innovative Resatorvid Topical Formulations for Targeted Skin Drug Delivery
Source: Pharmaceutics. 2022 Mar 24;14(4):700. doi: 10.3390/pharmaceutics14040700 (PMC9026853; doi:10.3390/pharmaceutics14040700)
Supplement: Supplementary file 1 [file pharmaceutics-14-00700-s001.zip › pharmaceutics-1549490 suppl.pdf]

Article

Design, Physicochemical Characterization, and In Vitro Permeation of Innovative Resatorvid Topical Formulations for Targeted Skin Drug Delivery

Victor H. Ruiz[1], David Encinas-Basurto[1], Bo Sun[1], Basanth Babu Eedara[1,2], Sally E. Dickinson[3,4], Georg T. Wondrak[1,3], H-H. Sherry Chow[3,5], Clara Curiel-Lewandrowski[3,6,7] and Heidi M. Mansour[1,2,7,8*]

[1] Department of Pharmacology and Toxicology, College of Pharmacy, The University of Arizona, Tucson, AZ 85721, USA
[2] Center for Translational Science, Florida International University, Port St. Lucie, FL 34987, USA
[3] Cancer Center, The University of Arizona, Tucson, AZ 85719, USA
[4] College of Medicine, Department of Pharmacology, The University of Arizona, Tucson, AZ 85724, USA
[5] College of Medicine, Department of Medicine, Division of Hematology and Oncology, The University of Arizona, Tucson, AZ 85724, USA
[6] College of Medicine, Department of Medicine, Division of Dermatology, The University of Arizona, Tucson, AZ 85724, USA
[7] BIO5 Institute, The University of Arizona, Tucson, AZ 85719, USA
[8]College of Medicine, Department of Medicine, Division of Translational & Regenerative Medicine, The University of Arizona, Tucson, AZ 85724, USA
* Correspondence: hmansour@fiu.edu; Tel.: +(772)-345-4731

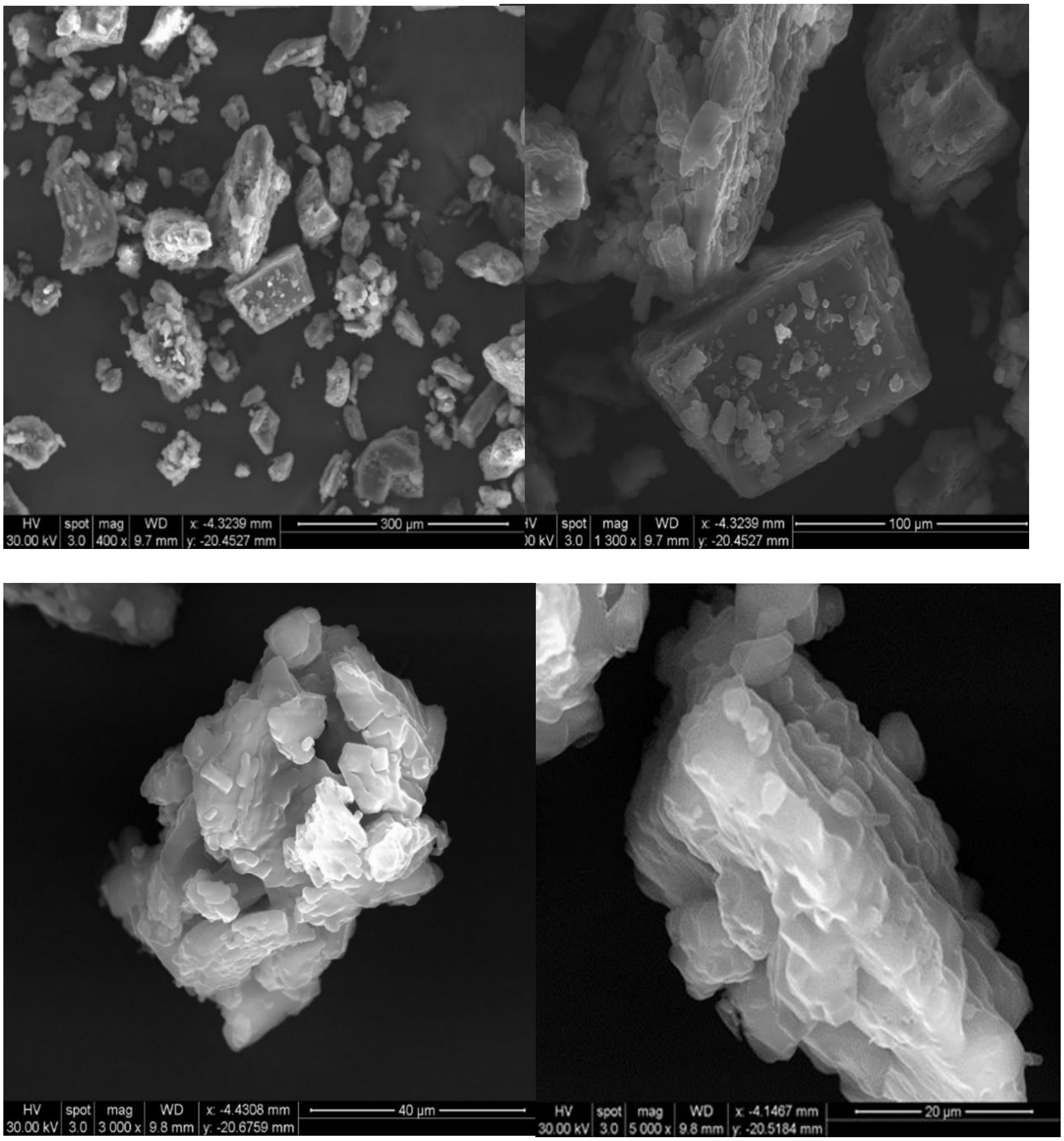

Figure S1. SEM micrographs of resatorvid at 400x, 1300x, 3000x and 5000x resolution.

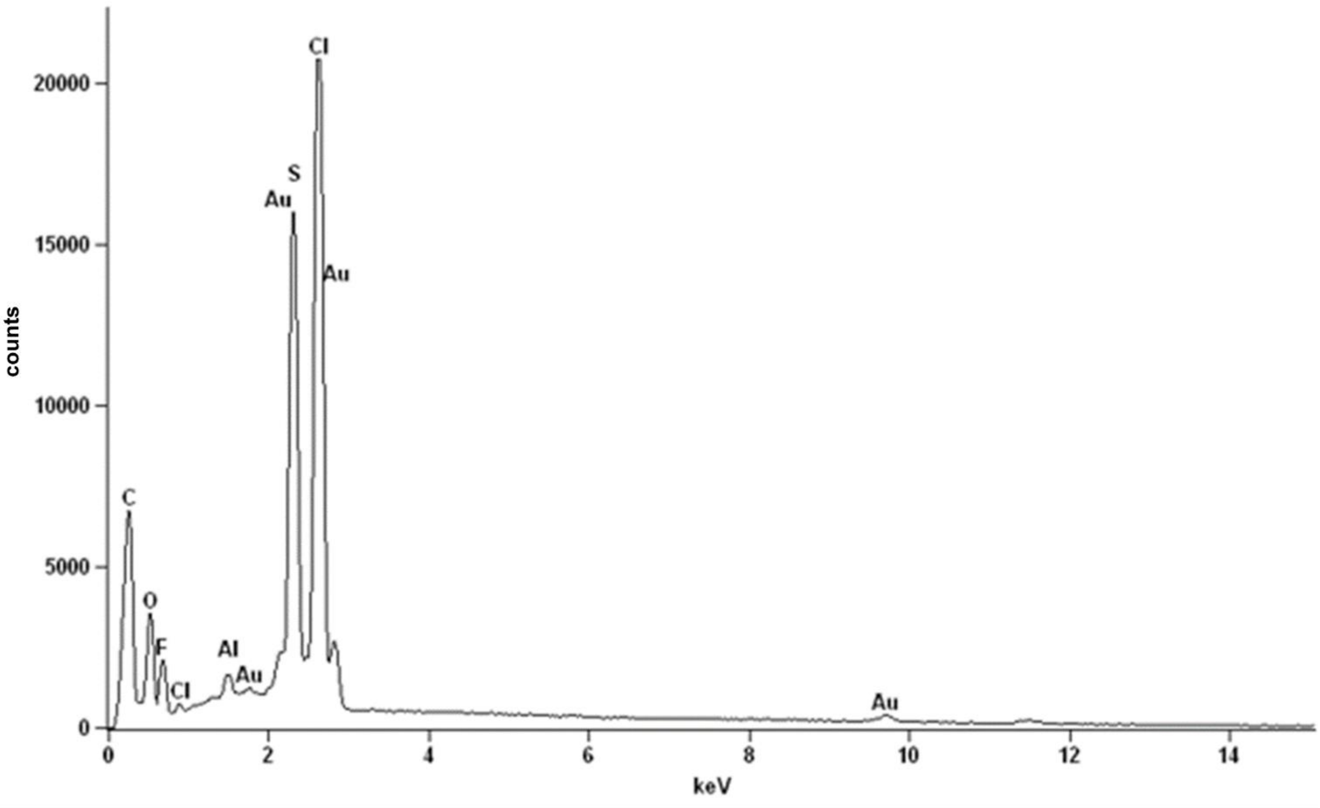

Figure S2. EDX spectra of raw resatorvid powder showing characteristic peaks. C-Carbon, O-Oxygen, S-Sulfur, F-Floride, and Cl-Chlorine atoms.

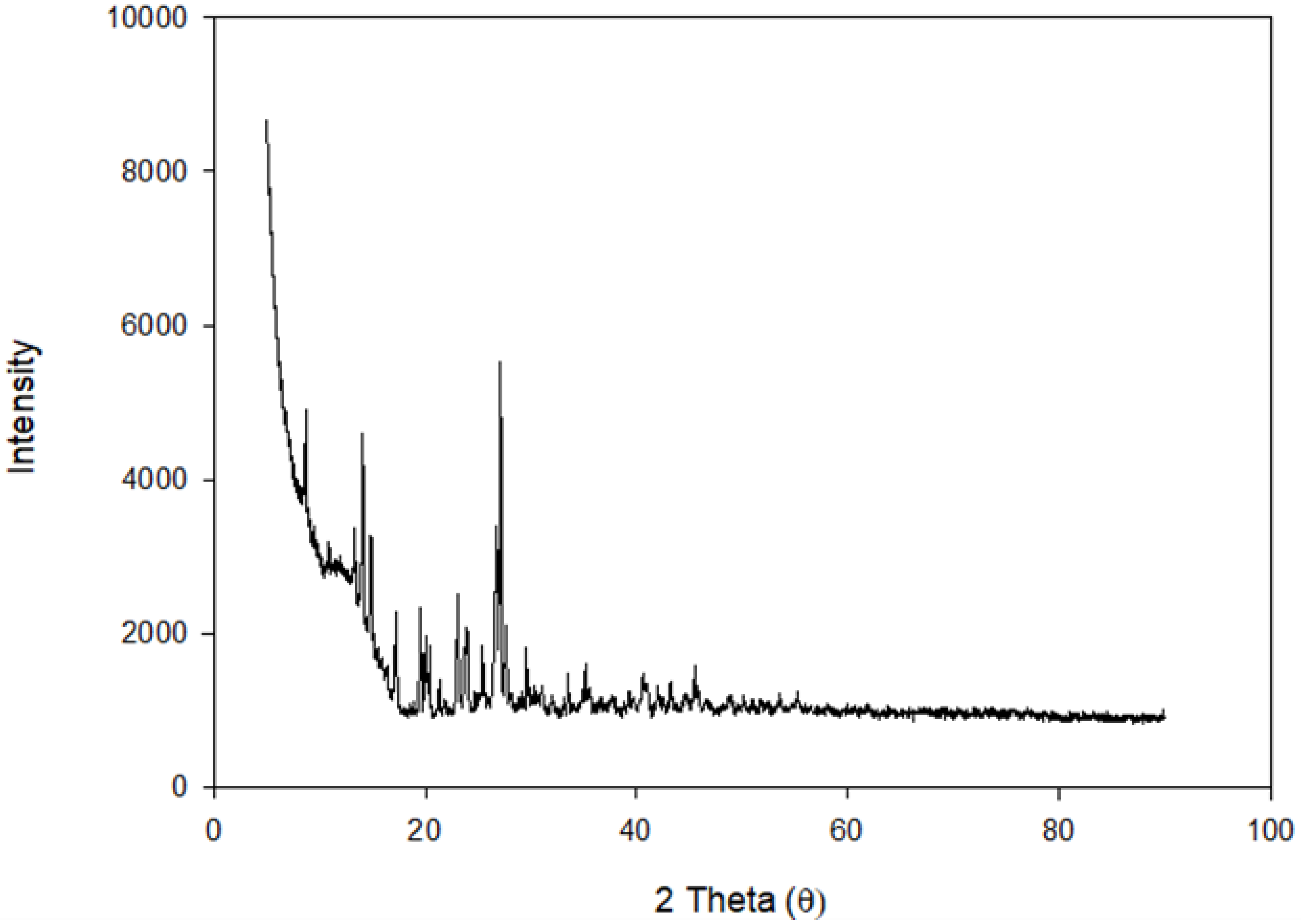

Figure S3: XRPD diffraction patterns of raw resatorvid.

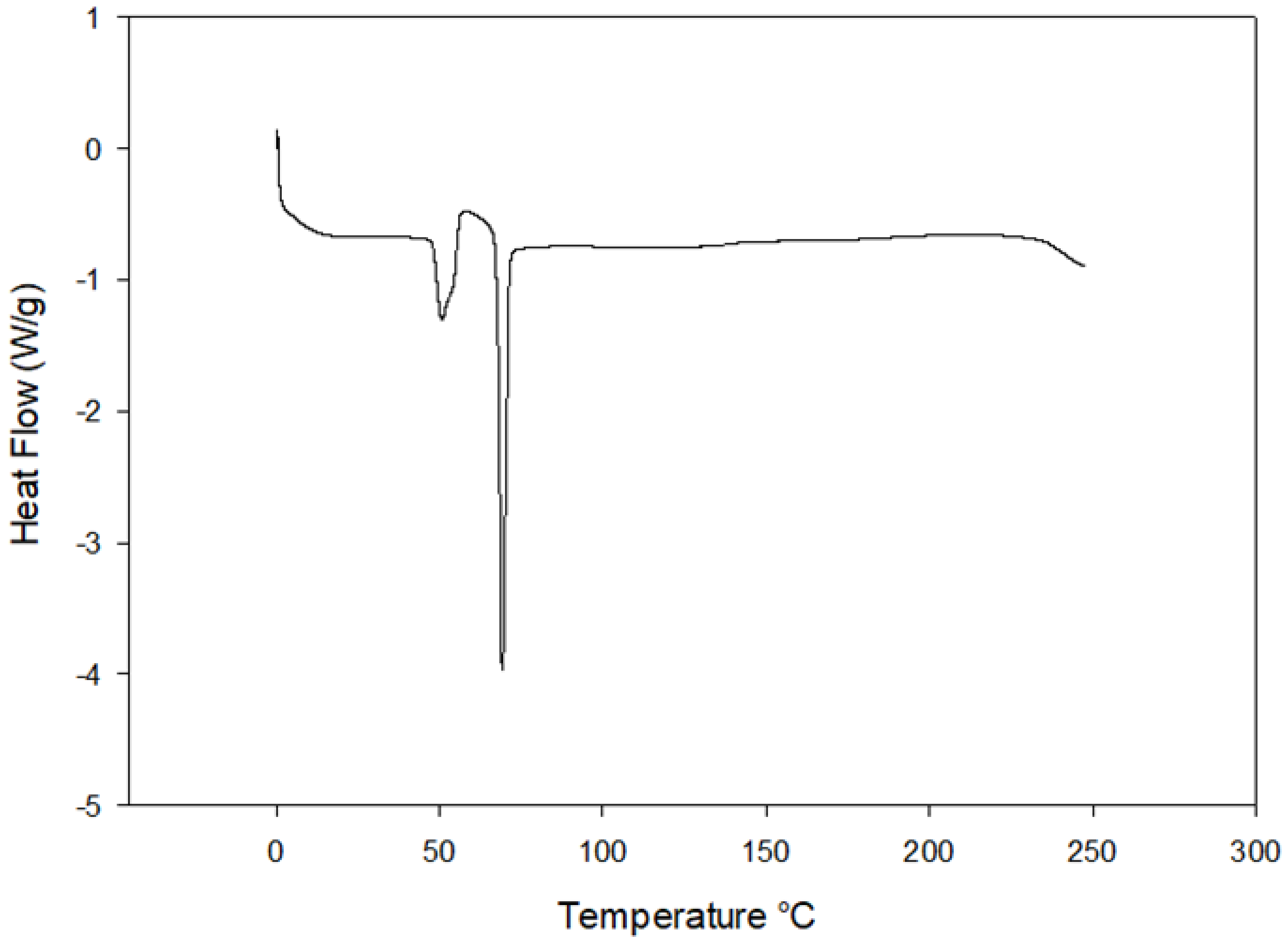

Figure S4. DSC thermogram of raw resatorvid.

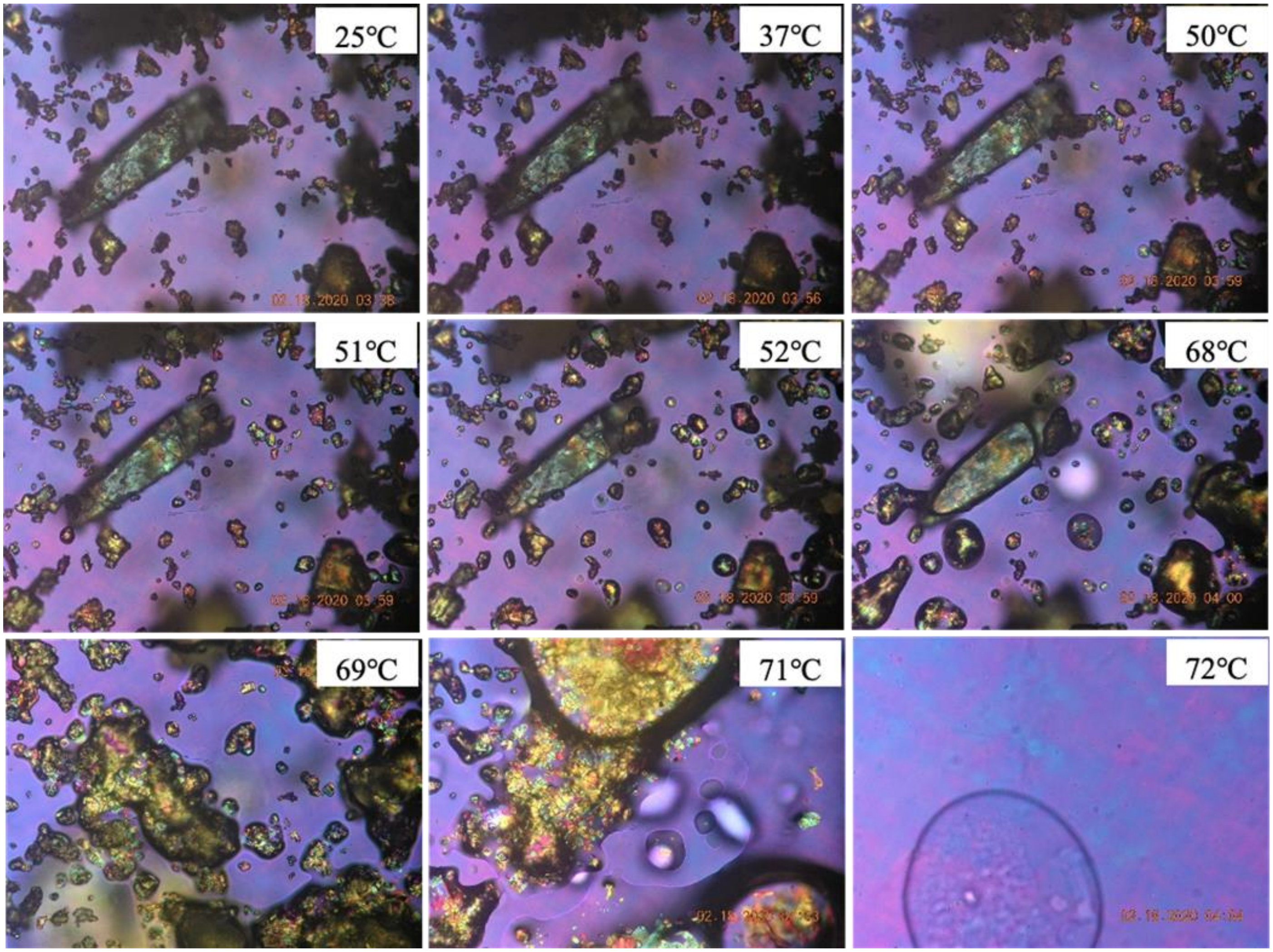

Figure S5. Representative HSM images of raw resatorvid.

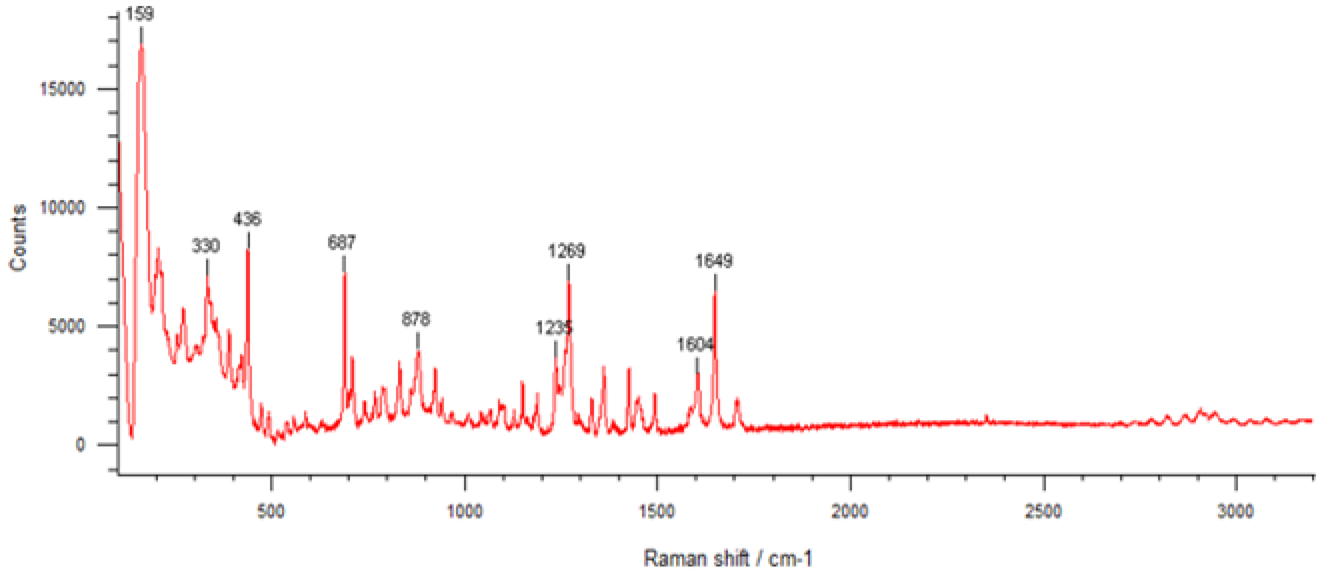

Figure S6. Representative Raman spectra of raw resatorvid using 785 nm laser.

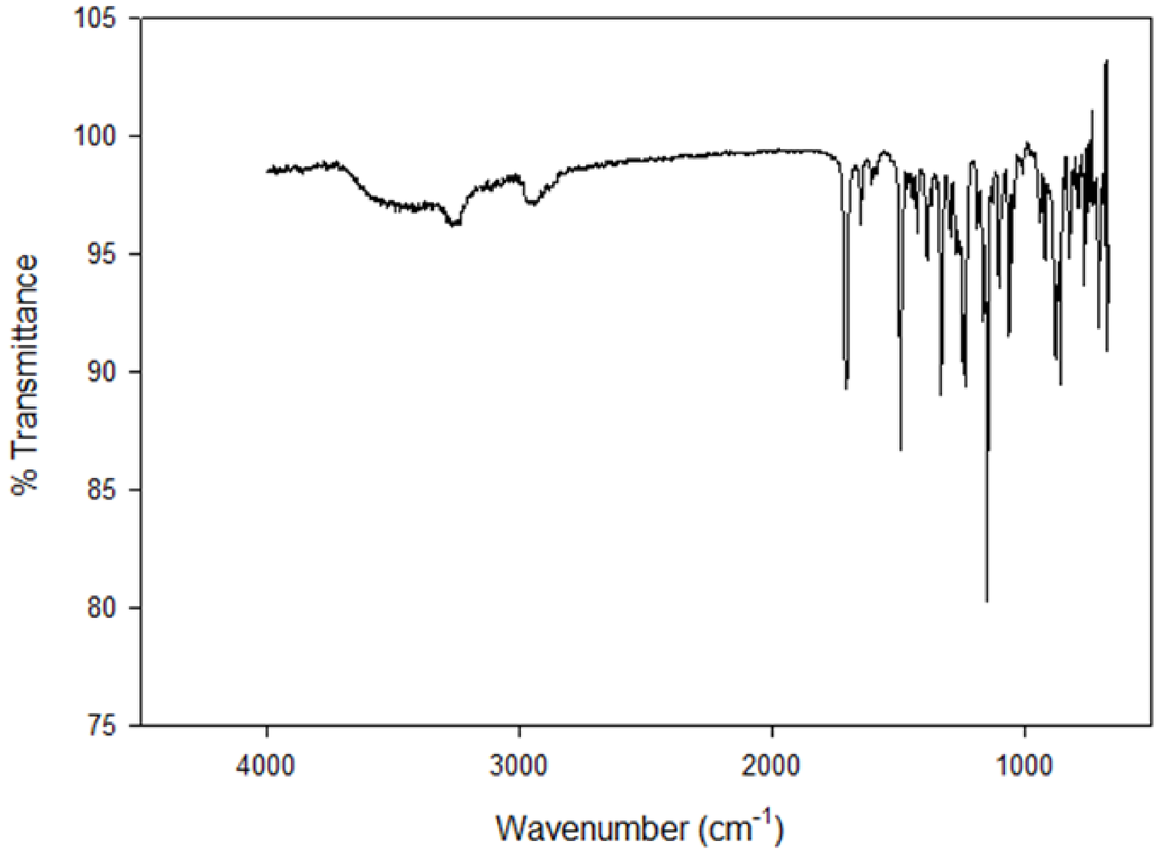

Figure S7. ATR-FTIR spectrum of raw resatorvid.

Table S1. DSC thermal analysis (n = 3, mean ± standard deviation).

	Resatorvid			
	Endotherm 1		Endotherm 2	
**System**	**T_{peak} (ºC)**	**Enthalpy (J/g)**	**T_{peak} (ºC)**	**Enthalpy (J/g)**
Raw Resatorvid	52.5±1.89	24.1±3.31	69.3±0.14	57.4±2.71

Table S2. Residual water content quantified by KFT. (n = 4, mean ± standard deviation).

Sample Identification	Residual Water content (%*w/w*)
TAK-242	0.0173 ± 0.0156
